# Recent Advances in the Fabrication and Application of Electrochemical Paper-Based Analytical Devices

**DOI:** 10.3390/bios14110561

**Published:** 2024-11-20

**Authors:** Zarfashan Shahid, Kornautchaya Veenuttranon, Xianbo Lu, Jiping Chen

**Affiliations:** 1 CAS Key Laboratory of Separation Science for Analytical Chemistry, Dalian Institute of Chemical Physics, Chinese Academy of Sciences, Dalian 116023, China; zarfashanshahid@dicp.ac.cn (Z.S.); kornautchaya@dicp.ac.cn (K.V.); chenjp@dicp.ac.cn (J.C.); 2 University of Chinese Academy of Sciences, Beijing 100049, China

**Keywords:** electrochemical paper-based analytical devices (ePADs), microfluidic paper-based analytical devices (μPAD), biosensors, clinical diagnosis, food analysis, environmental monitoring

## Abstract

In response to growing environmental concerns, the scientific community is increasingly incorporating green chemistry principles into modern analytical techniques. Electrochemical paper-based analytical devices (ePADs) have emerged as a sustainable and efficient alternative to conventional analytical devices, offering robust applications in point-of-care testing, personalized healthcare, environmental monitoring, and food safety. ePADs align with green chemistry by minimizing reagent use, reducing energy consumption, and being disposable, making them ideal for eco-friendly and cost-effective analyses. Their user-friendly interface, alongside sensitive and selective detection capabilities, has driven their popularity in recent years. This review traces the evolution of ePADs from simple designs to complex multilayered structures that optimize analyte flow and improve detection. It also delves into innovative electrode fabrication methods, assessing key advantages, limitations, and modification strategies for enhanced sensitivity. Application-focused sections explore recent advancements in using ePADs for detecting diseases, monitoring environmental hazards like heavy metals and bacterial contamination, and screening contaminants in food. The integration of cutting-edge technologies, such as wearable wireless devices and the Internet of Things (IoT), further positions ePADs at the forefront of point-of-care testing (POCT). Finally, the review identifies key research gaps and proposes future directions for the field.

## 1. Introduction

The current state of sensing technologies is still predominantly restricted to centralized laboratories, limiting their broader applicability and effectiveness in early diagnosis across fields such as healthcare, environment monitoring, and food safety. In resource-limited regions, delays in laboratory testing can significantly impact outcomes [1,2]. The recent pandemic has highlighted the necessity of rapid self-testing point-of-care testing (POCT) devices, which are essential for managing future global health crises [3]. Additionally, in response to increasing environmental concerns, the scientific community is progressively embracing the integration of green chemistry principles with modern analytical techniques [4].

Paper-based analytical devices (PADs) are now widely recognized as a viable solution for POCT applications and an influential technology across various fields. Their significant contributions to minimal reagent usage, reduced energy requirements, and biodegradability make PADs an ideal choice for sustainable and efficient analytical methods [5,6,7]. The fundamental working principle of PADs lies in the capillary action of paper [8], which enables passive fluid transport without external pumps or power sources, making them particularly useful for low-resource settings. The versatility of PADs stems from the use of paper as a substrate. Paper is thin, lightweight, available in various thicknesses, easy to store and transport, biocompatible with biological samples, and cost-effective. Paper also facilitates easy printing, coating, modification, and cutting, which make it an ideal substrate for numerous applications in analytical device fabrication. Additionally, its biodegradability, coupled with its effectiveness in analyte separation and filtration, significantly enhances its overall utility [9,10,11,12].

Several quantitative analytical methods have been investigated in the literature on paper-based POCT devices, employing techniques such as colorimetry, electrochemiluminescence, fluorescence, and electrochemical detection. Among these, electrochemical detection stands out as a particularly robust, cost-effective, and precise technique that offers rapid response times, exceptional sensitivity, selectivity, portability, and the ability for a multiplexed analysis. However, traditional electrochemical sensing approaches consume significant amounts of samples and reagents and require bulky potentiostat, microelectrodes, and other apparatus [13,14]. The incorporation of electrochemical sensing systems with PADs provides a unified platform for rapid, cost-effective, and multiplexed POCT analysis. This interdisciplinary approach has resulted in a new kind of plastic-free analytical device known as electrochemical paper-based analytical devices (ePADs), which successfully balance technological advancement and green chemistry principles to provide an efficient and environmentally friendly solution [15,16,17,18]. Since then, ePADs have significantly expanded their capabilities in target detection across diverse applications by using distinct electrochemical transduction methods, including amperometry [19], potentiometry [20], electrochemical impedance spectroscopy (EIS) [21], voltammetry such as cyclic voltammetry (CV) [22], differential pulse voltammetry (DPV) [23], and square wave voltammetry (SWV) [24].

**Figure 1:**
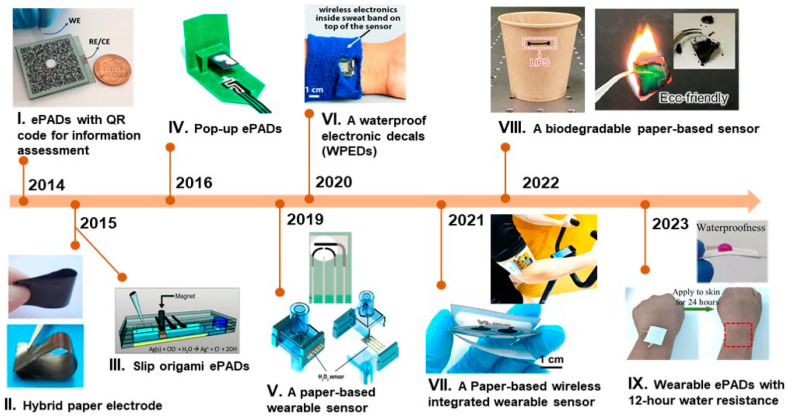
The technological timeline of the selected ePADs. (**I**) ePADs for p-nitrophenol determination. Reproduced from ref. [30]. Copyright (2014), with permission from Elsevier. (**II**) Pt nanoparticles enhanced graphene–carbon nanotube hybrid paper electrode. Reproduced from ref. [31]. Copyright (2015), with permission from Elsevier. (**III**) ePADs for detection of ricin at picomolar levels. Reproduced from ref. [32]. Copyright (2015), with permission from Royal Society of Chemistry. (**IV**) A pop-up ePADs for beta-hydroxybutyrate analysis. Reproduced from ref. [33]. Copyright (2016), with permission from the American Chemical Society. (**V**) A paper-based wearable sensor for hydrogen peroxide determination. Reproduced from ref. [34]. Copyright (2019), with permission from the American Chemical Society. (**VI**) A waterproof electronic decal for pH monitoring. Reproduced from ref. [35] Copyright (2020), with permission from Elsevier. (**VII**) A wireless wearable ePAD for sweat analysis. Reproduced from ref. [36]. Copyright (2021), with permission from Elsevier. (**VIII**) A laser-induced graphene ePADs for food spoilage monitoring. Reproduced from ref. [37]. Copyright (2022), with permission from Elsevier. (**IX**) A paper-integrated hydrophobic and air permeable piezoresistive sensors. Reproduced from ref. [38]. Copyright (2023), with permission from Springer Nature.

Recent developments in ePADs include integrating wearable technology, the Internet of Things (IoT), and automation to realize fully automated and robust devices that have brought new breakthroughs in the design of ePADs. However, the fabrication of cutting-edge ePADs with consistent and high-quality analytical performance involves numerous commercial hurdles that must be overcome in future advancements. In recent years, numerous studies on PADs have been documented, encompassing a broad spectrum of analytical techniques [25], as well as, on ePADs specifically focused on clinical applications [26,27,28,29]. However, unlike others, this review critically examines the progress in ePADs (Figure 1) for diverse applications in clinical diagnosis, food analysis, and environmental monitoring, encompassing a range of configurations from basic 2D to sophisticated multilayer 3D designs, and their impact on analyte flow control. Additionally, our paper highlights the integration of cutting-edge technologies like wireless wearable devices and the Internet of Things (IoT), underscores the significant advancements in the field, provides a SWOT analysis, and identifies critical research gaps and future directions.

In Figure 1, we showcase a selection of exemplary ePADs that have been made accessible over the past decade. For example, ePADs with integrated quick response (QR) codes for information tracking, first introduced in 2014, demonstrate the potential for efficient field testing (Figure 1I). The integration of QR codes allows users to efficiently collect and save relevant data about each assay on a portable electronic device. In 2015, researchers presented an innovative example of freestanding hybrid paper made of graphene–CNT that was modified with densely packed Pt nanoparticles (Figure 1II). This study demonstrated the material’s potential application for real-time amperometric sensing of H_2_O_2_. In the same year, researchers demonstrated the use of ePADs for detecting ricin in scenarios involving biological weapons (Figure 1III). In the origami Slip (oSlip) ePAD for quantitative detection of ricin, the sensor employs quantitative electrochemical detection of silver nanoparticle labels attached to a magnetic microbead support through a ricin immunosandwich assay. In 2016, a 3D pop-up paper device for measuring β-hydroxybutyrate, compatible with a commercial glucometer, was developed (Figure 1IV). Three-dimensional pop-up techniques, which create functional fluidic paths through folding, enhance the versatility of ePADs. Several ePADs have been integrated into wearable formats (Figure 1V–VII,IX), offering significant opportunities to enhance their functionality. Moreover, in response to green technology, researchers have developed a paper-based biodegradable temperature sensor, representing a fully green electronic solution (Figure 1VIII).

In this review, we also explore electrode fabrication and modification techniques that enhance multiplexed analysis and reaction kinetics, explore various electrochemical detection methods, and assess the integration of wearable technologies with IoT in ePADs. Our discussion aims to provide insights into the path towards the development of smart, sustainable, and highly effective ePADs. In addition, we present our perspective on the essentials of improving ePADs as well as an evaluation of future directions.

## 2. Fabrication of ePADs

**Figure 2:**
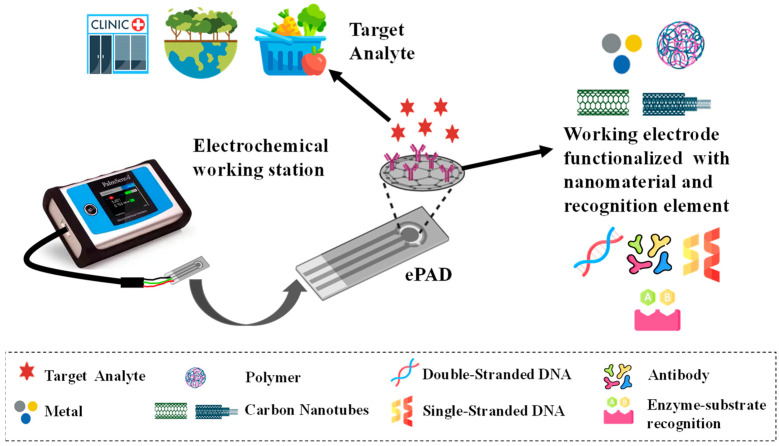
Visual representation of common ePAD components.

In general, the simplest ePADs consist of a substrate, hydrophobic channels for sample flow, an electrode system for detection, and an electrochemical workstation. The selection of a substrate requires consideration of several factors, including wicking rate, porosity, cost, and impurity resistance. After selecting the paper type, hydrophobic channels are fabricated to outline the sample and detection zones. The hydrophobic layer enables solution flow through capillary action. Moreover, it is crucial to ensure compatibility between these channels and the intended solution to prevent any potential interference, as the method of fabrication directly influences the fluid flow within the system [39,40,41]. Furthermore, the fabrication of ePADs involves configuring and assembling the device, followed by electrode fabrication, modification, and the functionalization of electrodes with various desired micro/nanomaterials, catalysts, binding molecules, and recognition elements. Figure 2 provides an overview of the main components in a typical ePAD.

### 2.1. Three-DimensionalePAD Configuration for Controlled Flow Dynamics

The foldable nature of paper greatly impacts the design and fabrication of ePADs. These devices can be categorized as either 2D, allowing for horizontal analyte flow across the x and y planes within a single paper layer, or 3D, enabling horizontal and vertical movement across the x, y, and z planes within a multilayer design [42,43]. The 3D design outperforms the 2D design in various aspects. It excels in the execution of multistep assays, as well as various pre-processing steps simultaneously and rapid detection with minimal sample volumes on a single device [44,45,46]. In 2D devices, the use of non-uniform porous paper for sample distribution and containment can lead to non-specific analyte absorption. This can result in inconsistent control over the analyte volume and disruptions within the electroactive zone, thereby affecting the overall sensor accuracy. On the other hand, 3D devices allow for customization of each layer in order to accommodate different chemical reactions, facilitating the seamless transition of the resulting product to the next layer and ensuring a uniform electroactive area across electrodes through multiple paper layers [47,48]. Currently, four methods are recognized for producing 3D ePADs. The first method involves stacking 2D layers of paper. This technique entails the assembly of alternating sheets of paper, which are typically bonded using double-sided adhesive tape. The sheets are intricately patterned to guide the fluid flow within and between the layers, thereby creating a three-dimensional structure [49]. The second method involves folding a single sheet of 2D paper into an origami shape. This technique integrates all components, such as channels, electrodes, and a sample zone, onto a single sheet. The final device is then folded along predefined creases, doubling over itself, and sealed with either a clamp or double-sided adhesive tape [50,51]. The third configuration is inspired by the design of pop-up greeting cards that transform from a 2D paper to a 3D structure by using a flap strategy. In the open format, the reagent and sample zones of the pop-up ePAD stayed distinct and separate. Upon closure of the device, a pathway for analyte flow is established as usual [52]. The fourth method involves rotational multilayer 3D ePADs. This configuration features multiple rotating disks with microfluidic pathways, enabling finer fluid flow manipulation through adjustable rotation speeds and times [53].

**Figure 3:**
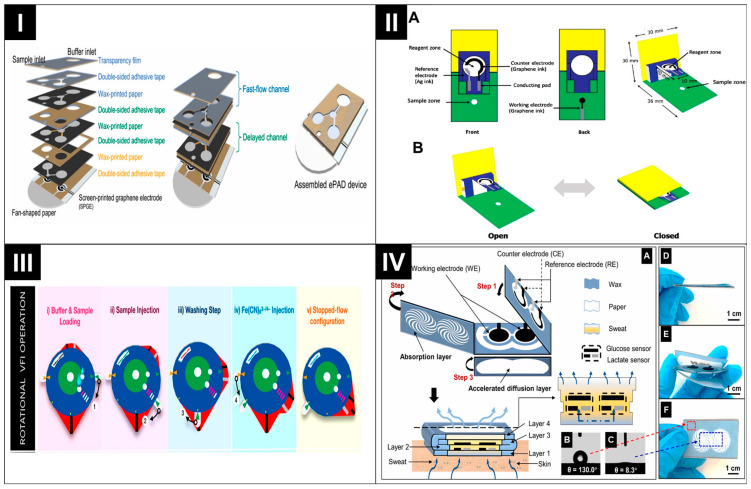
(**I**) Detailed illustration of the stacked ePAD. Reprinted with permission from ref. [55]. Copyright 2021, Elsevier. (**II**) Section of a pop-up ePAD (**A**), pop-up ePAD in the “open” and “closed” formats (**B**). Reprinted with permission from ref. [56]. Copyright 2020, Elsevier. (**III**) Schematic design of the rotational VFI ePAD. Reprinted with permission from ref. [53]. Copyright 2022, the American Chemical Society. (**IV**) Structural framework of origami HIS paper (**A**), evaluation of contact angles (**B**,**C**). Images displaying the foldability of device (**D**–**F**). Adapted with permission from ref. [36]. Copyright 2021, Elsevier.

The 3D multi-layered structure facilitates the controlled addition of multiple reagents, optimizes the flow dynamics of sample solutions through the paper, and ensures precise control over incubation time. This controlled process contributes to heightened sensitivity and accuracy in sensor performance [45,54]. For example, stacked 3D ePADs utilize paper layers to manipulate the volume capacity and multiple functionalities. A notable instance is a 3D ePAD designed for detecting hepatitis B and C which was developed by layering three sheets of wax-printed paper and double-sided adhesive tape onto a screen-printed graphene electrode (SPGE) printed on transparent film [55]. As shown in Figure 3I, the device is held together using an alligator clip. In this ePAD, each layer of paper serves a distinct purpose, including an inlet channel for sample entry, a rapid-flow channel for the automatic cleansing of free antigens, and a delayed channel for storing a redox reagent for subsequent analysis.

ePADs are also applicable in immunosensors and DNA sensors. For instance, Srisomwat et al. used a pop-up ePAD for label-free hepatitis B virus DNA detection [56]. Figure 3II illustrates the device’s composition, including a reagent and sample zone, a three-electrode system, and a conducting pad. The pop-up fabrication design ensures precise control of fluid flow, a target incubation time, and electrical connectivity; it also simplifies the sample introduction, reduces contamination risk, and minimizes exposure to the analyte. Similarly, Yakoh et al. developed an easy-to-use rotational vertical-flow immunosensor (VFI) ePAD that intentionally eliminates the interference of convective fluid motion. This ePAD allows users to conduct immunoassays by manually rotating the paper disk to sequentially transfer, switch, and stop fluid flows, as shown in Figure 3III, simplifying reagent delivery and completing the electroimmunoassay in just 9 min [53].

The integrated sensing paper, designed to detect multiple targets, has also been found applicable for use in wearable platforms. Li et al. developed the multi-layer origami ePAD for the wearable multiplexed analysis of sweat. As shown in Figure 3IV, the final device consists of four layers, each assigned a specific function, such as sweat collection, transfer, glucose and lactate sensing, and sweat evaporation [36]. These layers underwent a folding process, resulting in a final four-layer 3D structure. This design was intended to create a pathway for sweat diffusion by connecting the hydrophilic sections of the different layers. The purpose of this pathway was to collect and vertically diffuse sweat down the paper. Furthermore, the autonomous positioning of electrodes on different layers facilitated enzyme immobilization, enabling dual-channel multiplexed detection. Similarly, Cao et al. incorporated a 3D five-layer origami PAD (oPAD) into a skin-worn wearable device [57]. This design ensured a fresh liquid flow across the electrodes and prevented sweat accumulation within the device through a dedicated sweat-evaporating layer.

**Figure 4:**
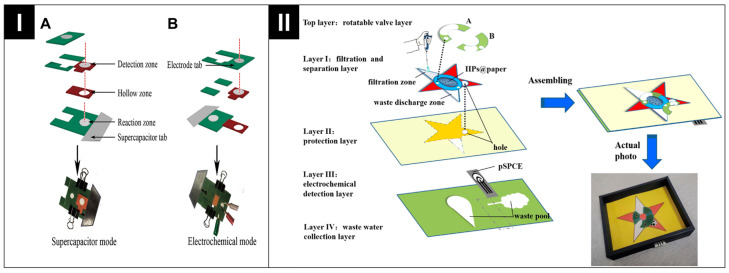
(**I**) Schematic illustration of the constructed pop-up oPAD (supercapacitor mode (**A**) and electrochemical mode (**B**)). Reprinted with permission from ref. [52]. Copyright 2020, Elsevier. (**II**) Detailed schematic design of an innovative 4-layer stacked ePAD design and assembly, alongside final assembly. Reprinted with permission from ref. [58]. Copyright 2024, Royal Society of Chemistry.

ePADs with dual configurations signify a notable advancement. These devices integrate multiple operational configurations within a single platform, harnessing synergetic effects that enhance operational sophistication and performance. Liu et al. applied both origami and pop-up designs to create a self-powered 3D ePAD [52]. Figure 4I illustrates the device’s composition, which involves an oPAD featuring various sections, including an electrode tab, a detection and reaction zone, a hollow area, and a supercapacitor tab on a single filter paper sheet. The pop-up strategy was used to spatially separate the detection zone and the reaction zone, ensuring no fluid connection before analysis and facilitating dual-mode operation (from supercapacitor to electrochemical mode) by modifying fluid connectivity and simplifying control over target incubation time. Hu et al. developed an innovative four-layer stacked ePAD that incorporates two rotating valves [58]. As shown in Figure 4II, the top layer features rotatable valves that precisely control liquid flow and incubation time by connecting or disconnecting specific channels on the paper layers. This setup allows for selective cadmium (Cd II) adsorption onto imprinted recognition sites, followed by efficient elution or separation to enhance sensor performance. Meanwhile, layer I streamlines filtration and separation, layer II protects, layer III enables electrochemical detection, and layer IV handles waste collection. Table 1 summarizes all key parameters of the state of the art pertaining to recent ePADs.

### 2.2. Integration of Diverse Paper and Other Substrate in 3D ePADs

**Figure 5:**
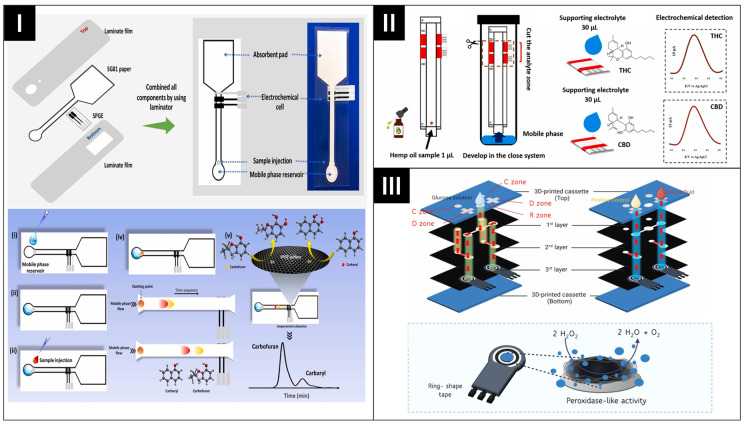
(**I**) Illustration of a separation-based ePAD for concurrent sensing of CBF and CBR. (i) Mobile-phase injection, (ii) the mobile phase flows to the detection zone by capillary action, (iii) sample injection, and (iv) the oxidation currents at the retention time of each carbamate pesticide are collected. Reprinted with permission from ref. [64]. Copyright 2023, Elsevier. (**II**) Schematic design of separation and quantitative analysis of THC and CBD using separation-based device. Reprinted with permission from ref. [63]. Copyright 2022, Elsevier. (**III**) Schematic visual of ePAD with two different papers as sample pad. Reprinted with permission from ref. [47]. Copyright 2024, Elsevier.

Recently, hybrid ePAD designs have been proposed to offer enhanced mechanical stability and biochemical analyses. In these devices, paper is often integrated with additional substrates to create the sample zone [61,62], facilitating the pretreatment and separation of analytes with similar chemical structures and redox potentials [63,64]. Kunpatee et al. engineered a separation-based ePAD for the real-time detection of carbofuran (CBF) and carbaryl (CBR) pesticides [64]. This ePAD utilizes a single SPGE as the working electrode and employs chromatographic paper to segregate the analytes based on their distinct partition coefficients (Figure 5I). This strategic approach effectively circumvents the issue of overlapping voltammogram peaks. Similarly, Pholsiri et al. used paper chromatography to separate Δ⁹-tetrahydrocannabinol (THC) and cannabidiol (CBD), followed by electrochemical measurement (Figure 5II) [63]. Recently, Permpoka et al. incorporated two types of paper with a commercial screen-printed electrode (SPE) to create a smartphone-enabled 3D oPAD [47]. The final device consists of three folded layers of filter paper within a 3D-printed cassette, secured with binder clips, as shown in Figure 5III. In this work, the nitrocellulose (NC) membrane is attached to the device’s initial layer of filter paper using a ring-shaped tape to immobilize the glucose oxidase (GOx), while the second and third layers are used to immobilize the antibodies and SPE placement, respectively.

Within the approach to combining paper with other substrates, Criscuolo et al. developed a hybrid ePAD for non-invasive lithium drug sensing [65]. This device integrates a flexible polyimide (PI)-based electrode with a paper fluidics system that features both fast- and slow-absorbing filter paper. This unique configuration is engineered to allow for the uninterrupted flow of fresh sweat onto the sensing zone while concurrently redirecting previously analyzed sweat to a separate reservoir. Caratelli et al. designed a 3D smart phone-assisted oPAD for pesticide analysis in the aerosol phase, comprising an office paper-based SPE and enzyme-infused filter pads, which enable pesticide detection by applying a few drops of the sample onto the enzymatic pad [60]. In contrast, Pokpas et al. integrated a pre-modified chromatographic paper disk into a commercial carbon SPE for metal analysis [66]. This 3D scaffold cell acted as both a storage medium for dry reagents and a reaction platform for the interaction of dimethylglyoxime, mercury (Hg), and electrolyte solution with nickel ions. Also, Cheng et al. developed a hybrid oPAD for cortisol detection, which integrates a commercial SPE with customized thread-based channels to precisely control sweat analyte flow and the sensor reaction sequence for optimal results [67].

### 2.3. New Method and Materials for Electrode Fabrication and Modification

#### 2.3.1. Electrode Fabrication

Electrode fabrication, including design, composition, and modification methods, greatly influence the performance of ePADs, affecting sensitivity, conductivity, overall performance, and the ability to conduct multiplex analyses. Since the pioneering research on ground-breaking research conducted by Dungchai, a range of methodologies for electrode fabrication has emerged, including screen printing, stencil printing, ink-jet printing, pencil drawing, sputtering, wire placing, and paper pyrolysis [42]. Table S1 offers a thorough summary, detailing the advantages, disadvantages, and concise descriptions of each technique and providing readers with a valuable resource for understanding each approach. The subsequent sections explore the latest advancements regarding new methods and materials for electrode fabrication that have emerged in recent years.

##### Porous Working Electrodes

Researchers are increasingly interested in porous working electrodes that can be fabricated using two primary methods: high-tech laser-scribing to produce high-quality porous graphitic films and simple coating techniques to apply conductive materials. These electrodes have superior active surface area-to-volume ratios and enhanced electron transport compared to traditional SPEs. They offer enhanced mass transfer from the bulk flow to the active sites, providing additional active sites and stronger convection transfer compared to planar electrodes [68,69,70]. However, the increased surface area, while beneficial for sensitivity, also makes porous electrodes more susceptible to fouling. Contaminants can block the pores, reducing the electrode’s effectiveness over time.

##### Conductive Paper as Electrodes

Conductive paper is manufactured by impregnating or coating paper substrates with conductive materials, such as conductive polymers or inks. Despite its advantages, achieving consistent conductivity and mechanical stability can be challenging. Recent advancements have demonstrated that dip coating and vacuum filtration are effective techniques for producing conductive paper electrodes. Dip coating involves immersing paper electrodes in conductive polymers or ink solutions, resulting in a uniform layer upon drying [71,72]. In contrast, vacuum filtration involves filtering a conductive material suspension through porous paper, resulting in a dense, deeply embedded conductive layer. These methods enhance the immobilization efficiency and electroanalytical properties of conductive paper-based electrodes [73,74].

##### Carbon Fiber Paper (CFP) Electrodes

CFP has attracted considerable interest as an electrode material owing to its remarkable characteristics. CFP, an electrically conductive material composed of over 90% carbon, is produced by laminating short, randomly arranged carbon fibers into a 2D sheet. This material is chemically inert, corrosion-resistant, and exhibits superior electrical properties, ensuring efficient electron transference. The key advantages of CFP include its high conductivity, flexibility, cost-effectiveness, and ease of processing. However, increased levels of heat treatment can decrease density, specific resistance, and tensile strength while improving conductivity. Compared to conventional screen-printed carbon electrodes (SPCEs), CFP electrodes offer improved performance, making them a valuable option for various electrochemical applications [75,76,77,78].

##### Microfluidic Electrochemical Carbon-Based Sensors (μCSs)

Recently, μCSs have emerged as a promising alternative to traditional SPCEs due to their enhanced sensitivity, cost-effectiveness, and potential for miniaturization. This technique commonly uses graphite foil as the base conductive material due to its chemical stability and high conductivity and applies electrodeposition and electropolymerization techniques for further nanomaterial modification. The electrodes are structured in a three-dimensional layout where the working and counter electrodes face each other across the microfluidic channel. This unique fabrication approach minimizes the distance between electrodes, promoting a more homogeneous electric field, which enhances analyte flow and overall sensor sensitivity. Despite these advantages, μCSs face challenges due to complex manufacturing processes and higher costs, which may limit their widespread use. Furthermore, although their three-dimensional design facilitates quick analysis, it requires additional optimization for large-scale implementation to guarantee practical viability [79,80,81].

#### 2.3.2. Electrode Configuration Methods for Multiplex ePADs

Multiplexing in ePADs can be achieved by integrating sensing elements onto a single working electrode, which enables the detection of multiple targets. This approach simplifies sensor design and reduces fabrication complexity. For example, a disposable ePAD was fabricated using boron-doped diamond (BDD) powder to quantitatively measure norepinephrine (NE) and serotonin (5-hydroxytryptamine, 5-HT) and to conduct anodic stripping voltammetry for heavy metals [82]. Similarly, a single gold electrochemical cell was utilized for detection of paracetamol and 4-aminophenol [83]. However, this multiplexing approach is effective only if the target analytes exhibit redox activity and possess distinctly different redox potentials.

**Figure 6:**
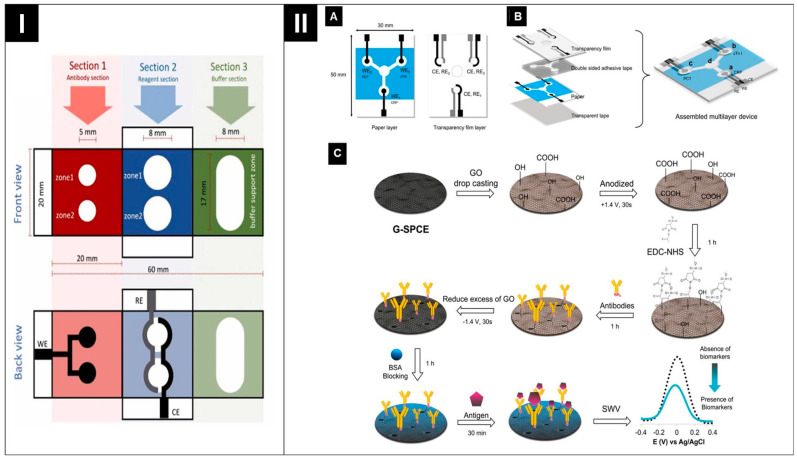
(**I**) Schematics of electrode fabrication: two separate WEs with a shared reference and counter electrode. Reprinted with permission from ref. [84]. Copyright 2024, Elsevier. (**II**) Schematic figure of multiple electrode cells integrated within a single device (**A**,**B**) and sequential modification and functionalization of the working electrode and identification of the antigen/biomarker on the sensor (**C**). Reprinted with permission from ref. [86]. Copyright 2021, Elsevier.

Recent advancements in multiplex ePADs involve either spatially separating the working electrode (WE) or incorporating multiple electrode cells within a single device. For instance, Chomthong et al. engineered an oPAD designed for the simultaneous detection of quinolone antibiotics [84]. This device features a multi-layer structure with a dual WE design, utilizing shared reference and counter electrodes, as illustrated in Figure 6I. Similarly, Boonyasit et al. developed a 3D ePAD for the simultaneous detection of diabetes markers. This device consists of two distinct screen-printed working and counter electrodes surrounded by a shared reference electrode [85]. Also, Boonkaew et al. developed a label-free ePAD for the multiplexed immunosensing of three cardiovascular disease biomarkers. As illustrated in Figure 6II, this device uses carbon SPE, which is further enhanced with graphene oxide (GO) and functionalized with different antibodies on each WE to target specific analytes [86]. These configurations allow each electrode to be individually tailored for the detection of specific analytes, enabling effective simultaneous analysis of multiple targets without interference. Additionally, separate working electrodes facilitate the customization of optimal conditions for detecting each specific target analyte through separate sample zones.

#### 2.3.3. Modification of Working Electrode and Its Effect on ePAD Performance Metrics

The working electrode, also known as the sensing or redox electrode, facilitates redox reactions on its surface, serving as the transduction element in ePADs. Modifications to the working electrode can significantly impact the overall functionality of the ePAD, particularly in terms of sensitivity, selectivity, and response speed. The choice of modification method is influenced by the redox behavior of target analytes as well as considerations such as application requirements, material properties, cost considerations, and functionalization needs. Nanomaterials are particularly advantageous for electrode modifications due to their exceptional properties, including large surface areas, enhanced catalytic activity, and reduced charge-transfer resistance [86,87]. Various nanoparticles (NPs) and composites of noble metal NPs (e.g., Cu, Ni, Au, Pt, and Ag), carbon NPs, metal–organic frameworks (MOFs), and metallic oxide NPs have been widely applied to enhance the working electrodes [87,88,89]. These materials also provide anchorage sites for immobilizing various recognition elements, including antibodies, aptamers, and DNA, thereby increasing their binding capacity and mobility [90,91].

The working electrodes can be modified through several techniques, including directly incorporating nanoparticles in ink [92], in situ growth of nanoparticles on the electrode surface [93], electrodeposition [94], drop-casting [95], and electropolymerization to embed nanoparticles onto the electrode surface [78]. These modification methods offer significant advantages, such as eliminating the need for enzymatic amplification, enhancing the peak separation of analytes with identical redox potentials [91], and improving the detection of target analytes that lack inherent redox activity through nanoparticle labeling [96]. In non-enzymatic ePADs, nanoparticle-modified electrodes also enable rapid electron transference at lower voltages, significantly reducing the oxidation potential required to catalyze the analyte reaction [97] compared to traditional electrodes which require higher voltages [98].

**Figure 7:**
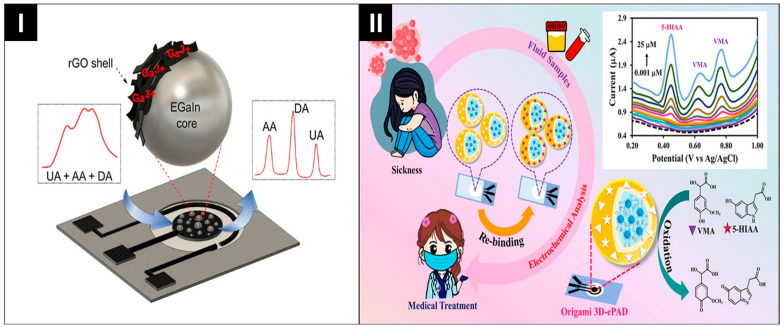
(**I**) Peak separation on a single electrode. Reprinted with permission from ref. [99]. Copyright 2021, the American Chemical Society. (**II**) Multiplexed assay on single electrode. Reprinted with permission from ref. [100]. Copyright 2024, Elsevier.

Recently, numerous studies on electrode modification have significantly enhanced the performance and versatility of electrochemical sensors. Scida et al. employed the magnetic preconcentration of silver nanoparticles (AgNPs) labels at a working electrode, achieving an impressive 250,000-fold signal amplification [91]. Baharfar et al. developed an SPE modified with reduced graphene oxide (rGO) over gallium–indium liquid metal droplets (EGaIn LM–rGO) which successfully distinguished the overlapping peaks of ascorbic acid, dopamine, and uric acid (Figure 7I) [99]. Ma et al. designed a 3D oPAD using Au Nanorod-modified paper working electrodes (AuNRs-PWEs) as the sensor platform. They used Au/bovine serum albumin (Au/BSA) nanospheres, coated with metal ions, as tracing tags for the concurrent multiplex detection of the non-redox active biomarkers CEA and CA125 [96].

Further functionalization of electrodes through the immobilization of recognition elements like peptide probes, aptamers, antibodies, and enzymes enhances the selectivity and sensitivity of ePADs. However, there has been a recent shift towards label-free detection methods in ePADs, incorporating unconventional elements such as molecularly imprinted polymers (MIPs). MIPs, which are artificial antibodies designed to replicate natural recognition entities, are particularly effective for creating specific recognition sites tailored to target molecules. Compared to conventional recognition elements, such as nucleic acids and antibodies, MIPs offer significant advantages in terms of stability, cost, and ease of preparation [101,102,103].

Recently, Somnet et al. developed a 3D oPAD that utilizes a single SPGE modified with graphene quantum dots (GQDs) and dual-molecularly imprinted polymers (GQDs@dual-MIP) for the simultaneous analysis of cancer biomarkers [100]. As shown in Figure 7II, this modification led to a pronounced surge in peak current, with a 3.5-fold enhancement for 5-Hydroxyindole-3-acetic acid (5-HIAA) and an 8-fold enhancement for vanillylmandelic acid (VMA) compared to the bare SPGE.

Another challenge in electrode modification for ePADs is electrode fouling, which significantly impacts sensor sensitivity, accuracy, and operational lifespan. This phenomenon occurs when biological or organic materials accumulate on the electrode’s active sensing surface, creating a barrier that impedes electron transfer and signal transduction. Such fouling compromises the quality and reliability of measurements and necessitates frequent electrode replacement or maintenance, thereby increasing operational costs and complexity. Addressing electrode fouling is, therefore, critical for advancing the practical applications of ePADs in real-world diagnostics and analytical processes. To combat this, researchers have developed various antifouling strategies. For instance, Nicoliche et al. developed in situ polysorbate 20 (T20) nanocoating to provide a protective barrier that prevents biofouling while maintaining sensor sensitivity [104]. Similarly, an alcohol-triggered capillarity was developed to enhance sample penetration and increase the electroactive surface area, reducing fouling through better sample integration [105]. In another example, Shan et al. employed tetrahedral DNA nanostructures, which offer robust antifouling capabilities and are particularly useful in biological fluid analysis [106]. Additionally, plasma treatments, such as oxygen plasma, improve surface hydrophilicity and cleanliness, preventing fouling and ensuring consistent sensor performance [107].

## 3. Detection Methods

Electrochemical techniques are a group of analytical methods that evaluate electron- transfer mechanisms, dynamics, concentrations of electroactive species, offer insights into detailed chemical kinetics, and provide comprehensive, reproducible characterization data [108,109,110]. The primary electroanalytical techniques used in ePADs include amperometry [19], potentiometry [20], EIS [21], and forms of voltammetry, such as CV [22], DPV [23], and SWV [24]. Among these techniques, voltammetry is the most widely used, accounting for 52% of detection methods, followed by impedance-based methods (28.9%), potentiometry (9.5%), amperometry (6.8%), conductometry (1.5%), and coulometry (1.2%) [111]. Various ePADs use different electrochemical detection techniques tailored to specific analytes, such as EIS for label-free biosensing [21,112] and potentiometry and voltammetry for the sensitive and simple analysis of organic pollutants and heavy metals [113,114].

Despite the availability of various electroanalytical techniques and compact digital glucose sensors, most ePADs still rely on bulky, expensive potentiostats that require trained professionals and a computer-based setup for operation, limiting their POCT applications in resource-limited settings. To address these limitations, researchers have developed portable, user-friendly miniature potentiostats with remote access and control capabilities for enabling autonomous operation.

For example, Fiore et al. developed a miniaturized, integrated circuit (IC)-based ePAD for amperometric cortisol detection. This portable ePAD converts analog electrochemical signals to digital using an onboard ADC (analog-to-digital converter), with data transmitted via Near-Field Communication (NFC) to a smartphone for real-time monitoring [115]. Cao et al. introduced a watch-integrated ePAD for potentiometric sweat analysis which utilizes ion-selective electrodes connected to an open circuit board. This device includes an ADC for signal processing, an onboard microcontroller that handles real-time data processing, and an OLED display for visualizing data for continuous monitoring [116]. Similarly, Liang et al. developed an oPAD alongside a wireless flexible printed circuit board (FPCB). This FPCB facilitates real-time monitoring of potassium levels in sweat during exercise. The circuit board includes IC components for potentiometric measurement and utilizes Bluetooth Low Energy (BLE) for wireless data transmission to a computer [117]. Given the current pace of advancements, it is reasonable to anticipate that compact ePADs with functional potentiostats, which provide diverse identification capabilities at a cost-effective price and intuitive usability for non-specialists, will soon become a reality.

## 4. Application of ePADs

ePADs present numerous advantages, including low cost, ease of modification, and portability, making them particularly effective for rapid detection applications. These attributes render ePADs invaluable for monitoring environmental contamination, ensuring food safety, and conducting medical analyses on a global scale, particularly in resource-limited areas.

### 4.1. Environmental Application

Rapid global industrialization and urbanization have led to the influx of various toxic and harmful substances into natural ecosystems, resulting in water contamination, air pollution, and severe health problems in humans [118]. Heavy metal ions, toxic chemicals, bisphenols, and pharmaceutical residues are hazardous environmental pollutants due to their high toxicity, carcinogenic properties, and low biodegradability [119,120]. Therefore, the detection of these pollutants at trace levels is essential for maintaining ecological safety and public health. Below are some examples of ePAD applications for environmental analysis.

**Figure 8:**
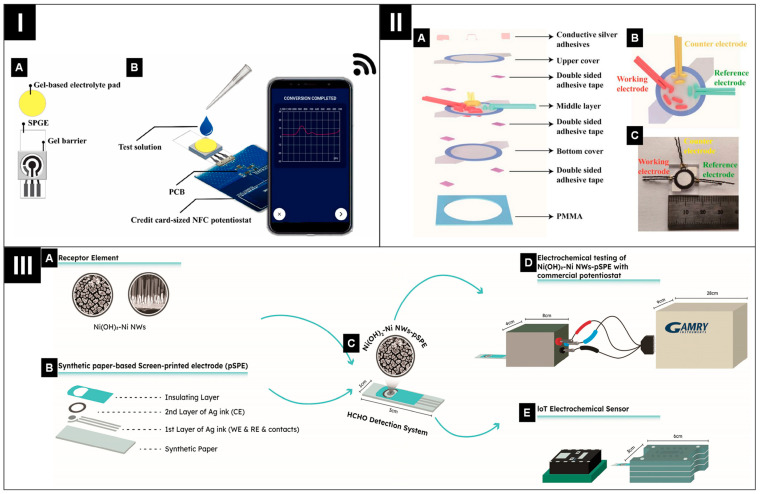
(**I**) Configuration of the gel-based ePAD (**A**) and its operational mechanism (**B**). Reprinted with permission from ref. [121]. Copyright 2024, Elsevier. (**II**) Schematic of the ePAD for H_2_O_2_ detection with its structure (**A**), top perspective of the central section (**B**) and image of the ePAD (**C**). Reprinted with permission from ref. [95]. Copyright 2024, Elsevier. (**III**) Schematic depiction of the IoT enabled ePAD, including SEM illustrations of the Ni(OH)_2__Ni NWs (**A**), the fabrication of pSPEs (**B**), the HCHO detection system (**C**), laboratory equipment (Gamry) (**D**), and IoT electrochemical setup (**E**). Reprinted with permission from ref. [123]. Copyright 2023, MDPI.

For instance, for multiplex heavy metal detection, a smartphone-enabled, paper-integrated wireless device was developed for simultaneous real-time sensing of Cd(II) and Pb(II) [121]. A pectin-based gel electrolyte formulated by dissolving pectin in a KCl solution mixed with an Sb(III)-Bi(III) bimetallic alloy solution was used; this mixture was cast onto a paper substrate, then precisely cut into disk shapes using a CO_2_ laser cutter before finally being incorporated into an SPGE, as shown in Figure 8I. This on-site pectin gel-based testing device exhibited two sharp and well-defined peaks with LOD of 50.98 ng mL^−1^ for Cd(II) and 40.80 ng mL^−1^ for Pb(II) via the differential pulse anodic stripping voltammetry (DPASV) technique, without any prior sample treatments.

Addressing potential health implications for humans, another study developed a reagent-free ePAD for hydrogen peroxide (H_2_O_2_) detection in real water samples [94]. They utilized a novel electrode fabrication approach by sewing electrodes onto paper substrates. As illustrated in Figure 8II, the final device consisted of three layers of paper, with carbon fiber electrodes sewn into the middle layer in a specific order using a needle, supported by a polymethyl methacrylate (PMMA) plate. The conductive silver adhesive was positioned on the fiber electrodes beyond the hydrophobic border to restrict the analyte within the platform. The ePADs demonstrated remarkable robustness against bending, exhibiting an error margin of only 6.2% after 20 bending cycles, and achieved an LOD of 0.9 µM. Expanding on reagent-free ePAD technology for soil analysis, Zeitoun et al. developed a reagent-free ePAD fabricated by the integration of a commercial SPE with a Mehlich-3 and molybdate-ion-impregnated filter paper reservoir [122]. The filter paper’s porosity enables the pre-loading of reagents into its cellulose network, serving as the site for phosphate extraction and the subsequent reaction. The ePAD has demonstrated its applicability for the rapid detection of phosphate in soil, showing an LOD of 0.11 mg L^−1^.

To address the detection of formaldehyde (HCHO), a significant water pollutant known for its toxicity, allergenic properties, and carcinogenic nature, an autonomous, portable, and IoT-enabled ePAD was developed for monitoring formaldehyde in water [123]. The device was fabricated by integrating Ni(OH)_2__Ni nanowires (NWs) onto synthetic paper-based screen-printed electrodes (pSPEs). The sensor platform includes a miniaturized potentiostat, Wi-Fi communication, and a Photon IoT development kit housed in a compact, protective case, as shown in Figure 8III. The practicability of this ePAD was tested by evaluating its ability to detect formaldehyde in both tap and distilled water. The Ni(OH)_2_Ni NWs/pSPE exhibits a quantification limit of 0.8 µM (24 ppb) of formaldehyde, with linear response across two concentration ranges: 0.8 to 500 µM and from 500 µM to 10 mM.

### 4.2. Food Analysis

Food safety is a significant concern because food pollutants have a dramatic impact on public health and social wellbeing [124]. These contaminants cover a range of foodborne hazards, including agricultural chemicals, mycotoxins, pesticides, dyes, and allergens. This section provides a systematic review of the latest developments in ePAD technology, focusing on their applications in the detection of chemical and biological hazards within the context of food safety.

**Figure 9:**
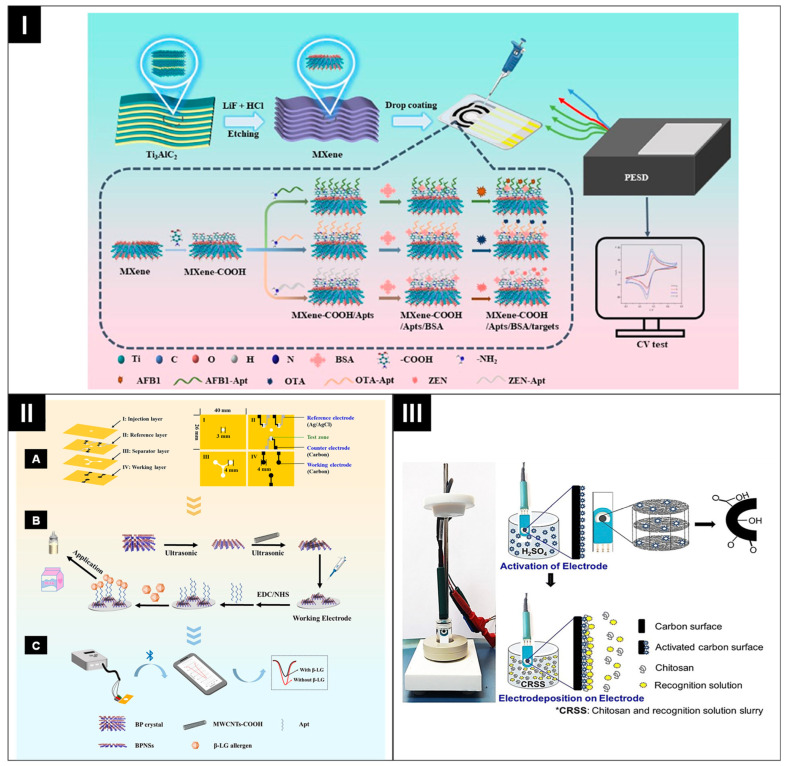
(**I**) Step-by-step fabrication method for MXene-based ePADs. Reprinted with permission from ref. [95]. Copyright 2024, Elsevier. (**II**) Fabrication of ePAD for milk allergen detection (**A**); the modification process of electrode and β-LG detection (**B**); handheld potentiostat for CV analysis (**C**). Reprinted with permission from ref. [128]. Copyright 2024, Elsevier. (**III**) A schematic representation of activation and electrodeposition of recognition solution on SPCEs for a dual-mode paper-based strip used in the detection of 4-DMAAB. Reprinted with permission from ref. [127]. Copyright 2024, Royal Society of Chemistry.

Mycotoxins, harmful secondary products of fungal metabolism, contaminate 25% of the global food supply and pose significant food safety risks throughout all stages of the supply chain [125]. Huang et al. have developed an advanced portable dual-mode paper chip that integrates electrochemiluminescence (ECL) and colorimetric detection for highly sensitive aflatoxin B1 (AFB1) detection [126]. The device consists of three main components: a colorimetric area, a liquid flow channel, and an ECL region, each mounted on polyvinyl chloride (PVC) substrates. In their study, a pretreated supernatant containing indicator molecules was drop-casted onto the CNT-modified electrode, which generated detectable signals. This paper chip demonstrates outstanding sensitivity, with an LOD of 7.8 fg mL^−1^ and a linear detection range from 50 fg mL^−1^ to 5 ng mL^−1^ for AFB1. For a multiplex analysis of mycotoxins, an MXene-based electrochemical aptasensor array (MBEAA) combined with a portable electrochemical detection device (PESD) was developed to detect multiple mycotoxins in corn [95]. The MBEAA was fabricated by laser-engraving three working electrodes with a shared reference and counter electrode on a hydrophobic paper tape template, which was then stuck to a PVC substrate. The working electrodes were modified by drop-casting an MXene solution and bio-functionalized by carboxylating the MXene surface to immobilize amino-labeled aptamers specific to AFB1, OTA, and ZEN, as shown in Figure 9I. The developed system demonstrated excellent analytical performance, achieving an LOD of 41.2 pg mL^−1^ for AFB1, 27.6 pg mL^−1^ for OTA, and 33.0 pg mL^−1^ for ZEN, with a detection range spanning from 0.1 to 10.0 ng mL^−1^.

Detecting pesticides is essential due to their extensive use, which has resulted in substantial environmental contamination and adverse effects on ecosystems and human health. Recently, Suk-in et al. developed a smartphone-enabled dual-mode (electrochemical and colorimetric) μPAD for the sensitive detection of carbaryl (CBR) in fruits [127]. They modified the SPGE with an AgNPs/MXene nanocomposite and integrated it with a PAD that included sections for colorimetric detection, electrochemical analysis, and CBR neutralization. Under optimized experimental conditions, the developed μPAD can detect CBR with a low LOD of 0.01 μM.

Milk and dairy products are a staple of the human diet. However, a significant proportion of the global population suffers from a milk protein allergy, which has become a global concern. Recently, Han et al. developed a 3D stack-up ePAD for milk allergen (β-lactoglobulin, β-LG) detection [128]. The device, as illustrated in Figure 9II, features a 3-electrode system on a 3D stack-up structure, where the working electrode is modified with black phosphorus nanosheets (BPNSs) and carboxylated multi-walled carbon nanotubes (MWCNTs–COOH) before being immobilized with β-LG specific aptamers. These aptamers bind the β-LG allergen, causing a conformational change that generates electrochemical signals. The practicability of the ePAD was tested by evaluating its ability to detect β-lactoglobulin in pure raw and powdered milk. The developed ePAD demonstrated an LOD of 0.12 ng mL^−1^ over a linear range of 10–1000 ng mL^−1^, closely aligning with the results from the standard HPLC method.

The detection of non-permitted dyes in food is also crucial due to their severe health risks, including mutagenic and carcinogenic effects. For instance, Rai et al. developed a dual-mode (electrochemical and chromogenic) paper-based strip for the detection of 4-(dimethylamino)-azobenzene (4-DMAAB) in mustard oil [129]. They fabricated an electrochemical sensor by modifying a SPCE. As illustrated in Figure 9III, the modification process involved a pretreatment to enhance the electrodes’ surface functionalities and hydrophilicity. Subsequently, the recognition elements and chitosan were deposited electrochemically. This approach improved sensor sensitivity and accuracy, achieving an LOD of 0.027 ± 0.008 mM with a linear range of up to 1.0 mM.

Artificial food antioxidants prevent oxidation and extend the shelf life of canned food. However, they also pose health risks, including toxicity and enzyme inhibition, making quantitative detection of these artificial antioxidants crucial for maintaining food safety. Recently, George et al. proposed an ePAD for artificial antioxidant butylated hydroxyanisole (BHA) detection [78]. An electropolymerization technique was employed to modify a CFP electrode with platinum nanoparticles (Pt NPs) on phosphorous-doped graphitic carbon nitride (PgCN). This CFP was subsequently modified with a molecularly imprinted polymer to enable customized BHA recognition. A key innovation in this study is the solvent-free electrochemical extraction method, which employs prolonged electrolysis instead of traditional chemical washing. The sensor achieved an impressive low LOD of 5.83 nM and displayed a linear detection range of up to 210 μM.

### 4.3. Medical Application

ePADs have transformed the field of medical and clinical analysis by offering low-cost POC diagnostic solutions. Their portability and ease of use are particularly valuable in areas lacking traditional laboratory infrastructure, providing crucial information for disease diagnosis, monitoring, and management. Furthermore, the adaptability and versatility of ePADs make them suitable for a variety of biomedical applications, from monitoring glucose levels in diabetic patients to detecting viral diseases like COVID-19. 

**Figure 10:**
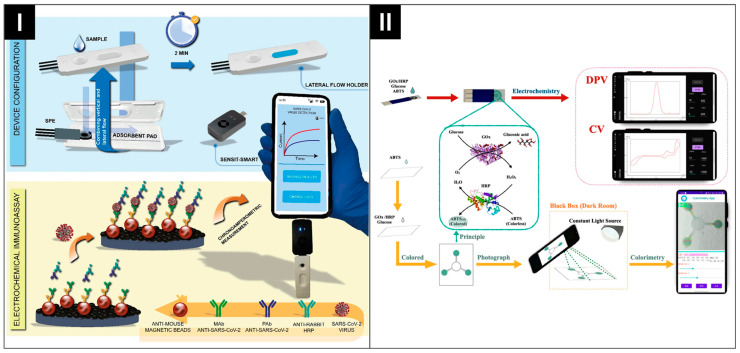
(**I**) Device configuration and modification for electrochemical detection of SARS-CoV-2. Reprinted with permission from ref. [130]. Copyright 2024, Elsevier. (**II**) Scheme of dual method to detect glucose, based on ABTS-ePAD. Reprinted with permission from ref. [23]. Copyright 2024, Elsevier.

Fabiani et al. created a smartphone-assisted, zero waste ePAD to detect SARS-CoV-2 in saliva samples, integrating both vertical and lateral flow strategies [130]. The ePAD comprised a compartment with all the necessary reagents for assembling the immunological chain on magnetic beads, as well as a lateral flow holder featuring a carbon black-modified SPE, a magnet, and a fluidic absorption paper pad. A magnetic bead-based immunological chain was prepared by immobilizing the capture and polyclonal anti-SARS-CoV-2 antibodies along with an HRP-labeled secondary antibody through 30 min incubation at ambient temperature without shaking (as shown in Figure 10I). Finally, the addition of a washing buffer and 3,3′,5,5′-tetramethylbenzidine (TMB) reagent to the holder initiated the analysis process of N-protein. The sensor achieves an LOD of 30 ng mL^−1^ and operates within a linear range of 0.06 to 4 μg mL^−1^.

Another example of a smartphone-enabled dual-mode system for medical application is the ePAD developed by Xu et al., which combines electrochemical and colorimetric methods for glucose detection [23]. The system, as shown in Figure 10II, integrates a hand-held detector with SPE modified with glucose oxidase (GOx) and horseradish peroxidase (HRP). This setup utilizes 2,2′-azino-bis (3-ethylbenzothiazoline-6-sulfonic acid) (ABTS) substrates to facilitate glucose analysis. Utilizing differential pulse voltammetry (DPV), the ePAD demonstrated an LOD of 0.467 µM and a linear response from 1 to 500 µM.

An example of a flexible, low-cost electrochemical immunosensor is the device fabricated by Sweety et al., which utilizes conducting paper to detect the cancer antigen epithelial cell adhesion molecule (EpCAM) [71]. The conducting paper is developed by dip-coating it in CuS-modified PEDOT: PSS and then doping it with dimethyl sulfoxide (DMSO) to enhance its conductivity. For the immunosensor assembly, anti-EpCAM antibodies are immobilized on the modified paper and bovine serum albumin (BSA) is applied to prevent non-specific adsorption. The practicability of the sensor is evaluated using human serum. This immunosensor demonstrated a wide linear detection range from 0.01 pg mL^−1^ to 1000 ng mL^−1^. In a similar vein, Raucci et al. developed a sustainable device using a chromatographic paper-based disk and SPE on office paper for preconcentration and electroanalytical determination of miRNA-652 associated with triple-negative breast cancer (TNBC) [131,132,133]. To enhance selectivity and sensitivity, the SPE was further modified with gold nanoparticles (AuNPs) and an anti-miRNA probe tagged with methylene blue (MB). Under optimized conditions, the sensor achieved an LOD of 0.4 nM and demonstrated a linear response of up to 800 nM in both standard solutions and human serum.

### 4.4. Wireless and Wearable ePAD Applications

The accelerated growth of the global population, the increasing prevalence of chronic illnesses, and the emergence of viral pandemics have created a pressing need for innovative solutions that can effectively address the urgent demands regarding public health and safety in healthcare systems. The latest developments in healthcare technology have focused on the fabrication of autonomous systems capable of continuous monitoring of physiological parameters anywhere and anytime. Paper substrates for wearable electronics provide a sustainable, cost-effective, and flexible alternative to plastic-based wearables. They enhance sensor accuracy, reduce biofluid buildup, and offer comfortable, breathable options for long-term use that are ideal for elderly and mobility-limited individuals [134].

Recent advances in sensor miniaturization and wireless communication technologies, such as ZigBee [135], radiofrequency identification (RFID) [134], NFC [135], and BLE [136], have significantly contributed to this shift. Bluetooth and NFC are especially favored for wireless sensing for data transmission due to their compatibility with smartphones and software. NFC can be powered wirelessly by smartphones, reducing power consumption and enhancing practical applications. However, NFC is limited by a short transmission span of just a few centimeters, whereas Bluetooth supports data transfer over distances of up to approximately 10 m [136,137,138,139,140,141,142]. These technologies enable the seamless integration of ePADs with digital tools like smartphones, smartwatches, and tablets through the Internet of Things (IoT), allowing for real-time monitoring, continuous communication with professionals, quick emergency decision-making, and reduced diagnostic costs.

Wireless wearable ePADs have revolutionized patient monitoring and diagnostics in the biomedical field. These devices offer continuous, non-invasive monitoring of cortisol [117], glucose and lactate [36], potassium ion [117], and chronic wounds [141], as well as vital signs such as heart rate [139] and body temperature [140,143], providing real-time data that can be remotely accessed by healthcare providers. In addition, some advancements have been made in developing wireless ePADs for environmental monitoring and food analysis; for example, BPA detection [141], soil and environmental humidity [142], plant hormone detection [93], and food spoilage monitoring [37].

**Figure 11:**
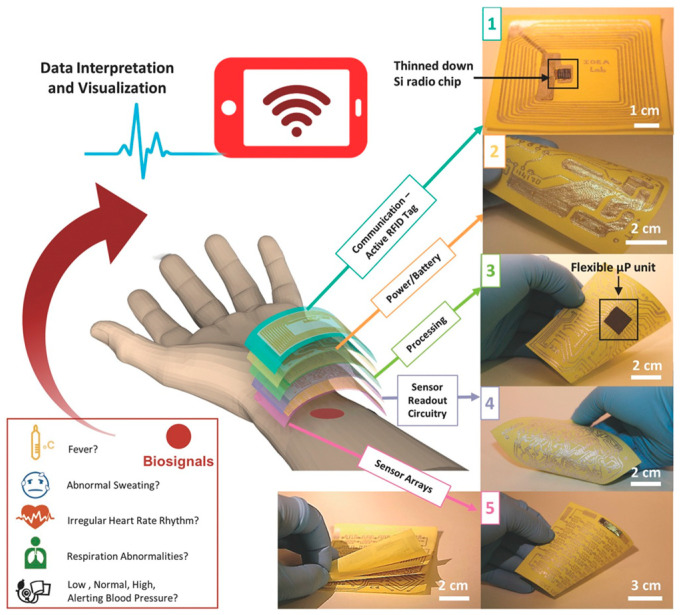
Conceptual demonstration of a fully autonomous, wearable, wireless, stacked 3D ePAD for healthcare monitoring. (1) Digital photo of an active RFID tag on paper with a flexible Si-based radio chip for wireless data communication. (2) Power source circuitry printed on paper. (3) Processing unit with a flexible Si-based microprocessor (μP) integrated via flip-chip with printed circuitry on paper. (4) Sensor’s readout circuitry fully printed on paper. (5) Multifunctional healthcare sensory platform. Reprinted with permission from ref. [143]. Copyright 2017, Wiley.

As demonstrated previously, numerous studies have been focused on the advancement of wireless wearable ePADs. However, only a few studies have comprehensively explored the fabrication of fully paper-supported wireless wearable devices. Marking a significant milestone in this direction, Nassar et al. pioneered a fully autonomous wearable wireless all-paper-supported system for data processing, power supply, and signal transmission [143]. The system incorporates a Post-it Note paper substrate, an RFID tag, power management features, a silicon microprocessor, and multiple sensors (as shown in Figure 11). The final paper watch monitors vital signs (blood pressure, body temperature, skin hydration, and heart rate) and transmits data directly to smartphone interfaces. However, this design employs Post-it Note paper, which comes with a thin layer of adhesive polymer that slightly compromises its breathability. In another recent study, Lou et al. developed an affordable, comfortable, and user-friendly sock-embedded wearable sensor for atrial fibrillation (AFib) monitoring [144]. A five-channel tactile sensor array was fabricated through spray-coating metallization. The sensor array, coupled with an energy-efficient application-specific integrated circuit (ASIC) and a Bluetooth low-energy (BLE) chip, is seamlessly incorporated into a compact wireless sensing system. This system is strategically designed for discreet placement on the upper part of the foot inside a sock. Evaluations conducted under different sitting conditions using a commercial electrocardiographic (ECG) chest band and a photoplethysmographic (PPG) sensor as benchmarks show that the sensor accurately measures heart rate variability (HRV) and instant heart rate (HR), accurately detecting AFib during normal foot movements while the user is seated. Results also indicate the detection of supraventricular tachycardia (SVT). While the sock-embedded AFib monitor represents a significant innovation, its sensor performance could be compromised during intense foot movements.

Even though numerous proof-of-concept demonstrations exist, the commercial adoption of these devices is still in its infancy, except for glucose monitors, which highlight a significant research gap in both fundamental and applied aspects. Extensive research is required to enhance the sensitivity, selectivity, long-term stability, reliability, and multiplexing capabilities of wireless wearable ePADs that consistently perform well with relevant analytes in real-world scenarios. Table S2 summarizes the applications and performance metrics of various ePADs across environmental, food, and medical analyses, providing a comprehensive comparison with existing literature to highlight advancements in detection capabilities and real-time adaptability.

## 5. Limitations and Challenges

Over recent years, significant advancements have been made in the development of ePADs. ePADs are highly regarded for their user-friendliness, affordability, and rapid diagnostic capabilities, which have facilitated their application across a diverse range of fields. Despite these advancements, the progression and widespread commercialization of ePADs have encountered various limitations and challenges. These challenges are primarily categorized into the following areas: technical limitations, issues related to miniaturization, environmental constraints, and regulatory and commercialization barriers.

While ePADs demonstrate commendable sensitivity and specificity, they often fall short when compared to traditional market competitors, especially concerning accuracy, reproducibility, and sensitivity. Moreover, ePADs are highly susceptible to environmental constraints such as humidity, temperature variations, light exposure, mechanical stress, and electrode fouling, which can significantly alter their physical, chemical, and electrical properties. Consequently, these variations can impact the performance, stability, and shelf life of ePADs [38,118,145]. The miniaturization of ePADs and bulky potentiostat to portable pocket-sized readouts also presents a substantial challenge to developers, particularly in terms of ensuring that devices can maintain consistent quality without compromising sensitivity, specificity, or cost-efficiency. Further, the commercialization of ePADs, particularly in the healthcare industry, requires navigating complex regulatory processes to meet strict standards of clinical safety and reliability. Additionally, achieving market acceptance necessitates extensive clinical validation and educational initiatives to help stakeholders recognize the benefits of ePADs over traditional diagnostic tools [146,147].

**Figure 12:**
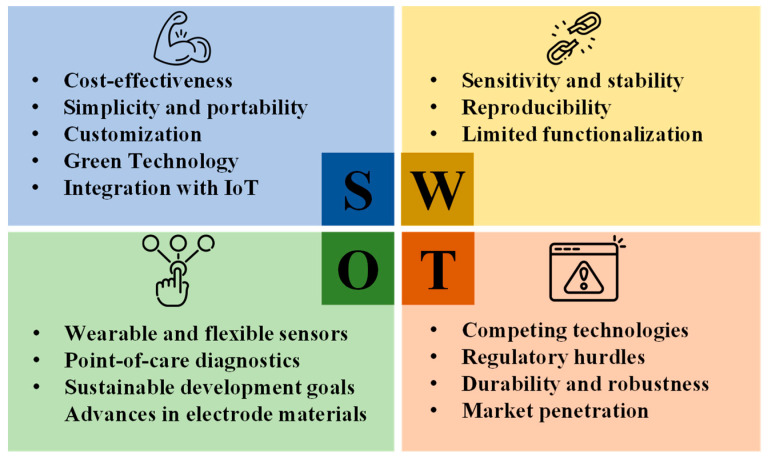
A SWOT analysis of thr current state of research and the future potential of ePADs.

These challenges are not merely obstacles; they also serve as gateways to innovation and growth. They emphasize the importance of strategic planning and management, enabling researchers and scientists to thoroughly evaluate the benefits, risks, opportunities, and challenges associated with project hypotheses in the field of ePADs. In addition, we have incorporated a SWOT analysis (Strengths, Weaknesses, Opportunities, and Threats) in Figure 12 to provide a clearer perspective on the current advancements and future potential of ePADs.

## 6. Conclusions and Perspective

ePADs are recognized as effective analytical tools for rapid, on-site testing, and their affordability, portability, and user-friendly design make them invaluable across various applications in healthcare, diagnostics, and environmental monitoring. Nanomaterials have enabled significant advancements and opened new possibilities for electrode designs and structures. Now, ePADs have emerged as strong competitors to conventional electrochemical sensors, distinguished by their incorporation of an autonomous microfluidic network that enhances fluid transport and enables sample treatment. Despite recent advancements, the reliance on expensive commercial equipment (working station) for ePAD analysis remains a challenge, necessitating the fabrication of economical and user-friendly ePADs for successful commercialization in disease diagnosis, food analysis, and environmental surveillance.

Further research is expected to achieve notable advancements in wireless, multiplexed, self-powered technology, and simplified assay procedures for successful long-term monitoring through non-invasive analysis and widespread public applications. The most significant challenge in the development of wearable ePADs is ensuring sufficient power. Lithium batteries are preferred for their longevity, but their substantial bulk presents significant drawbacks. Innovative approaches, such as energy harvesting from biofluid using biofuel cells and energy storage using electrochemical supercapacitors, might be an efficient and ideal approach. There is significant potential for further research in optimizing the use of biodegradable cellulose substrates while systematically reducing or eliminating non-biodegradable materials to reduce environmental impact. Additionally, automating testing processes in ePADs can significantly improve efficiency and accuracy, and there is still room for further research in the future.

To ensure the successful public deployment of ePADs, prioritizing user-friendliness is crucial. This includes easy use, comprehensive data interpretation, an enhanced user experience, tailored applications, and predictive outcomes through integrated algorithms. Interdisciplinary research partnerships could play a vital role in transforming the vision of a fully regenerative next-generation ePAD from concept to practical reality, advancing beyond mere research and development.

## Figures and Tables

**Table 1:** Detailed comparison with all the key parameters of recent ePAD.

ePAD Configuration	Electrode Fabrication	Electrode Material	Sample	LOD	Application	Ref.
Stacked and rotational	Screen printing	rGO/SPCE	Water samples	0.05 ng ml^−1^	Cadmium (II)	[58]
Origami	Screen printing	Ti_3_C_2_T_x_/PB/SPCE	Wound fluids	26.97 nM	Myeloperoxidase	[47]
Rotational	stencil-printing	GSPCE	Human serum	3.54 fg ml^−1^	α-fetoprotein	[53]
Origami	Screen printing	Mn–ZnS QDs @PT-MIP/GSPE	Fruit samples	0.2 nM	Patulin	[50]
Origami	Screen printing	AuNPs/PPy/SPCE	Human serum	0.654 pg ml^−1^	Interleukin-6	[59]
Flower-like origami	Screen printing	CB/PBNPs/SPCE	Aerosol phase	2 ppb	Paraoxon	[60]

SPCE = screen-printed carbon electrode, PB = prussian blue, GSPCE = graphene screen-printed electrode, Mn–ZnS QDs@PT-MIP = manganese-zinc sulfide quantum dots coated with patulin imprinted polymer, AuNPs = gold nanoparticle, PPy = polypyrrole, CB = carbon black.

## Data Availability

Not applicable.

## Supplementary Materials

The following supporting information can be downloaded at: https://www.mdpi.com/article/10.3390/bios14110561/s1, Table S1. Summarization of electrode fabrication techniques, including their pros and cons. Table S2. ePADs application with comprehensive performance metrics [19,23,37,50,69,95,121,123,130,143,148,149,150,151,152,153,154,155,156,157,158,159,160].
