# Supplementary material for: Recent Advances in the Fabrication and Application of Electrochemical Paper-Based Analytical Devices
Source: Biosensors (Basel). 2024 Nov 20;14(11):561. doi: 10.3390/bios14110561 (PMC11592294; doi:10.3390/bios14110561)
Supplement: Supplementary file 1 [file biosensors-14-00561-s001.zip › biosensors-3265345-supplementary.pdf]

Supporting information for:

Recent advances in fabrication and application of electrochemical paper-based analytical devices

Zarfashan Shahid [1,2], Kornautchaya Veenuttranon [1,2], Xianbo Lu [1,*], Jiping Chen [1]

[1] CAS Key Laboratory of Separation Science for Analytical Chemistry, Dalian Institute of Chemical Physics, Chinese Academy of Sciences, Dalian, 116023, P. R. China
[2] University of Chinese Academy of Sciences, Beijing 100049, P. R. China
* Correspondence: xianbolu@dicp.ac.cn (X.L.)

Table S1. Summarization of electrode fabrication techniques, including their pros and cons.

Fabrication method	Short description	Equipment and material	Advantages	Disadvantages	Ref.
Screen printing/ Stencil printing	Screen printing is a stencil-based procedure that divides the design into closed and open portions. It works by squeezing the viscous ink through open sections of the mesh onto the printing surface.	Customized nylon, silk, or woven mesh screens, inks	Affordable, easy to design, compatible with various inks, suitable for high volume production, and a wide range of commercial conductive pastes are available.	Required specific masks optimization of tailored ink composition is hectic and offer low resolution, high ink waste, require high viscosity ink and adhesion problems.	[1,2]
Sputtering/ Chemical vapor deposition	This procedure involves exposing the paper substrate to one or more volatile precursors, which then break down on the substrate surface to form the required thin film deposit.	Sputtering chamber and vacuum	Higher conductivity, uniformity of conductive tracks, homogeneous ink coverage.	Expensive, difficulties in mass-production, requirement of expensive equipment, and films.	[3,4]
Inkjet printing	Inkjet printing is a fully computerized process that creates a pattern by depositing thin layers of conductive	Inkjet printer and inks	High patterning capacity, could print substrates simultaneously via different	Nozzle blockage, expensive, high-tech tools requirement, ink	[5,6]

	material from precursor inks ejected through nozzles with pinpoint accuracy over a predetermined area and design.		ink, various substrate fabrication option, high reproducibility, no pre-deposit, and template required, suitable for mass production.	viscosity issue, and difficulty to balance the ink formulation.	
Wire placement	In this method, prefabricated wires are incorporated into the paper surface.	Wires, binders and tapes	Easily chemical cleaning, higher electrochemical performance, electrode could be modified without destroying the paper substrate.	Noble metal weirs are expensive, difficult to manage the electrode zone, and less electroactive area.	[7-9]
Pyrolysed paper	In this process, a tube furnace is used for the thermochemical treatment of paper in the absence of oxygen and a controlled atmosphere (95% Ar /5% H_2, at 1000 °C for 30 min). Further, paper is used as a working electrode on a solid base.	Tube furnace and gasses	Low resistivity, the verity of carbon precursors can be used.	Furnace and elevated temperatures, difficulties in the powder compressing and patterning.	[10-12]
Laser scribing of carbon materials	This process primarily relies on the use pyrolysis of carbon materials to produce controlled carbonaceous tracks using a commercial laser (mostly CO_2) with insulating features.	Commercial CO_2 laser	Inexpensive, suitable for large-scale yield, mask-free, one-step, and regent-free process.	Reproducibility relies on the type of laser power used, which is not suitable for all types of paper substrates.	[13,14]
Pencil/Pen drawing	In this simple fabrication, a pencil/pen is used to draw conductive electrodes on a piece of card or paper by hand drawing or an automatic plotter.	Pencils and commercial conductive ink pens	Quick fabrication, zero-waste process, inexpensive, no requirement for after-heat dealing, straightforward graphite transfer to paper, broad range commercial availability of pen and pencil.	Variation in commercial pencil composition, low reproducibility, poor control of graphite film thickness.	[15,16]

Table S2. ePADs application with comprehensive performance metrics

Environmental analysis					
**Target analyte**	**Fabrication Tool/Method**	**Working electrode material/Substrate**	**Detection method**	**Performance metrics**	**Comparison to Literature**
Heavy metal detection ePAD [17]	Screen printing	Sb–Bi/BiNP/SPGE in pectin-based electrolyte on filter paper	DPASV	Smartphone-enabled, real-time sensing for Cd(II) and Pb(II); LOD: 50.98 ng/mL for Cd(II), 40.80 ng/mL for Pb(II); no pre-treatment of sample needed	Demonstrates high sensitivity and yields analytical results that are closely aligned with those of the standard ICP-OES method, while offering superior adaptability for real-time applications.
H_2O_2 detection [18]	Needle sewing	PB/Carbon fiber electrode onto the paper and PMMA support	CA	Reagent-free, highly robust (only 6.2% error after 20 bending cycles); LOD: 0.9 μM	Offer competitive H_2O_2 detection capabilities and exhibits improved mechanical stability compared to similar reported ePADs.
Formaldehyde Detection ePAD [19]	Screen printing	$Ni(OH)_2$–Ni NWs-pSPE on paper	CA	IoT-enabled with miniaturized potentiostat; LOD: 0.8 μM	Competitive performance compared to existing ePADs and offers cost-effective portability, improving practicality for onsite real-time applications.

			**Food analysis**		
Multiplex mycotoxin detection [20]	Laser-engraving	Bio functionalized MXene-based carbon electrode array on paper tape template and pasted on the PVC plastic substrate	CV	Multiplex detection of three mycotoxins with miniaturized potentiostat ; LODs: 41.2 pg/mL for AFB1, 27.6 pg/mL for OTA, 33.0 pg/mL for ZEN	Improved multiplexing capabilities and results of real sample are comparable with traditional HPLC method and existing ePADs, offers portability and improved onsite detection with portable electrochemical simultaneous device (PESD).
Food spoilage monitoring [21]	Laser irradiation (LI)	LIG electrode on biodegradable commercial paper, milk carton and a meat price tag	Resistance measurement	Wireless real-time monitoring of thermo- and chemical- status of food, no need of further functionalization	Biodegradable and directly integrated into food packaging, functional without an embedded heater and better performance than existing sensors
			**Medical application**		
SARS-CoV-2 detection [22]	Screen printing	CB-SPE/MB on paper-based pad	Amperometry	Waste-free, combines vertical and lateral flow for sample and waste management, and smartphone-assisted for easy data handling; LOD: 30 ng/mL	Compared to traditional as RT-PCR method, this ePAD provides quick detection without the need for laboratory setup, demonstrates high sensitivity even at low viral loads, and features a user-friendly design

Glucose[23]	Screen printing	GOx/HRP/ABTS modified SPCE on chromatographic paper	DPV	Dual-mode detection: electrochemical and colorimetric, smartphone integration for data acquisition and processing portable and cost-effective; LOD: 0.467 μM	Demonstrates high sensitivity than existing sensors, User-friendly APP with a hand-held detector, Results closely align with the results from the standard method.
**Wireless and wearable ePAD**					
Wearable paper watch for vital signs [24]	Pen drawing	Silver ink-based sensor arrays on post-it note paper	Capacitive and resistive	Cost-effective, recyclable, easy fabrication, skin-conforming, no need of further functionalization, strong mechanical stability under bending.	One of the first all-paper-supported sustainable wireless wearable ePAD for continuous health monitoring,

Sb–Bi/BiNP/SPGE: Antimony–bismuth/bismuth nanoparticles/screen-printed graphite electrode; DPASV: Differential pulse anodic stripping voltammetry; ICP-OES: Inductively coupled plasma optical emission spectrometry; PB: Prussian blue; CA: Chronoamperometry; PMMA: Poly (methyl methacrylate) macromolecule; NWs: Ni nanowires; LIG: Laser-induced graphene; CB-SPE: Carbon Black-based screen-printed electrode; MB: Magnetic beads; SPCE: Screen-printed carbon electrodes; GOx/HRP/ABTS: Glucose oxidase/horseradish peroxidase/2,2-azino-bis (3-ethylbenzothiazoline-6-sulfonic acid)

References:

1. Sodkrathok, P.; Karuwan, C.; Kamsong, W.; Tuantranont, A.; Amatatongchai, M. Patulin-imprinted origami 3D-ePAD based on graphene screen-printed electrode modified with Mn–ZnS quantum dot coated with a molecularly imprinted polymer. *Talanta* **2023**, *262*, 124695.
2. de Oliveira, T.R.; Fonseca, W.T.; de Oliveira Setti, G.; Faria, R.C. Fast and flexible strategy to produce electrochemical paper-based analytical devices using a craft cutter printer to create wax barrier and screen-printed electrodes. *Talanta* **2019**, *195*, 480-489.
3. Shiroma, L.Y.; Santhiago, M.; Gobbi, A.L.; Kubota, L.T. Separation and electrochemical detection of paracetamol and 4-aminophenol in a paper-based microfluidic device. *Anal. Chim. Acta* **2012**, *725*, 44-50.
4. Kokkinos, C.; Economou, A.; Giokas, D. based device with a sputtered tin-film electrode for the voltammetric determination of Cd (II) and Zn (II). *Sensors Actuators B: Chemical* **2018**, *260*, 223-226.
5. Ray, A.; Mohan, J.M.; Amreen, K.; Dubey, S.K.; Javed, A.; Ponnalagu, R.; Goel, S. Ink-jet-printed CuO nanoparticle-enhanced miniaturized paper-based electrochemical platform for hypochlorite sensing. *Applied Nanoscience* **2023**, *13*, 1855-1861.
6. Bihar, E.; Wustoni, S.; Pappa, A.M.; Salama, K.N.; Baran, D.; Inal, S. A fully inkjet-printed disposable glucose sensor on paper. *npj Flexible Electronics* **2018**, *2*, 30.
7. Cheng, X.; He, C.; Zhang, W.; Wan, H.; Shi, Q.; Liu, H. Performance Testing of Paper-based Electrochemical Sensor for Blood pH Measurements. *International Journal of Electrochemical Science* **2022**, *17*, 221259.
8. Mettakoonpitak, J.; Boehle, K.; Nantaphol, S.; Teengam, P.; Adkins, J.A.; Srisa‐Art, M.; Henry, C.S. Electrochemistry on paper‐based analytical devices: a review. *Electroanalysis* **2016**, *28*, 1420-1436.
9. Fosdick, S.E.; Anderson, M.J.; Renault, C.; DeGregory, P.R.; Loussaert, J.A.; Crooks, R.M. Wire, mesh, and fiber electrodes for paper-based electroanalytical devices. *Analytical chemistry* **2014**, *86*, 3659-3666.
10. de Lima Tinoco, M.V.; Fujii, L.R.; Nicoliche, C.Y.; Giordano, G.F.; Barbosa, J.A.; da Rocha, J.F.; Dos Santos, G.T.; Bettini, J.; Santhiago, M.; Strauss, M. Scalable and green formation of graphitic nanolayers produces highly conductive pyrolyzed paper toward sensitive electrochemical sensors. *Nanoscale* **2023**, *15*, 6201-6214.
11. Silva, L.A.J.; da Silva, W.P.; Giuliani, J.G.; Canobre, S.C.; Garcia, C.D.; Munoz, R.A.A.; Richter, E.M. Use of pyrolyzed paper as disposable substrates for voltammetric determination of trace metals. *Talanta* **2017**, *165*, 33-38.
12. Giuliani, J.G.; Benavidez, T.E.; Duran, G.M.; Vinogradova, E.; Rios, A.; Garcia, C.D. Development and characterization of carbon based electrodes from pyrolyzed paper for biosensing applications. *Journal of Electroanalytical Chemistry* **2016**, *765*, 8-15.
13. Patella, B.; Parisi, A.; Moukri, N.; Gitto, F.; Busacca, A.; Aiello, G.; Russo, M.; O'Riordan, A.; Inguanta, R. Phosphate ions detection by using an electrochemical sensor based on laser-scribed graphene oxide on paper. *Electrochim. Acta* **2023**, *461*, 142600.
14. de Araujo, W.R.; Frasson, C.M.; Ameku, W.A.; Silva, J.R.; Angnes, L.; Paixão, T.R. Single‐step reagentless laser scribing fabrication of electrochemical paper‐based analytical devices. *Angew. Chem.* **2017**, *129*, 15309-15313.
15. Dossi, N.; Petrazzi, S.; Toniolo, R.; Tubaro, F.; Terzi, F.; Piccin, E.; Svigelj, R.; Bontempelli, G. Digitally controlled procedure for assembling fully drawn paper-based electroanalytical platforms. *Analytical chemistry* **2017**, *89*, 10454-10460.

16. Pagkali, V.; Soulis, D.; Kokkinos, C.; Economou, A. Fully drawn electrochemical paper-based glucose biosensors fabricated by a high-throughput dual-step pen-on-paper approach with commercial writing stationery. *Sensors Actuators B: Chemical* **2022**, *358*, 131546.
17. Lersanansit, N.; Pungjunun, K.; Chailapakul, O.; Praphairaksit, N. Development of pectin-based gel electrolyte for wireless electrochemical determination of cadmium and lead using smartphone. *Talanta* **2024**, *276*, 126211.
18. Wang, Y.; Ye, D.; Xu, Y.; Zhu, X.; Yang, Y.; Chen, R.; Liao, Q. An electrochemical paper-based analytical device with facile carbon fiber-sewed electrodes for highly sensitive detection of hydrogen peroxide in real water. *Electrochim. Acta* **2024**, *484*, 144091.
19. Trafela, Š.; Krishnamurthy, A.; Soderžnik, K.Ž.; Kavčič, U.; Karlovits, I.; Klopčič, B.; Šturm, S.; Žužek, K. IoT Electrochemical Sensor with Integrated Ni (OH) 2–Ni Nanowires for Detecting Formaldehyde in Tap Water. *Sensors* **2023**, *23*, 4676.
20. Wang, C.; Zhao, X.; Huang, X.; Xu, F.; Gu, C.; Yu, S.; Zhang, X.; Qian, J. Simultaneous detection of multiple mycotoxins using MXene-based electrochemical aptasensor array and a self-developed multi-channel portable device. *Talanta* **2024**, *278*, 126450.
21. Jung, Y.; Min, J.; Choi, J.; Bang, J.; Jeong, S.; Pyun, K.R.; Ahn, J.; Cho, Y.; Hong, S.; Hong, S. Smart paper electronics by laser-induced graphene for biodegradable real-time food spoilage monitoring. *Applied Materials Today* **2022**, *29*, 101589.
22. Fabiani, L.; Fiore, L.; Fillo, S.; D'Amore, N.; De Santis, R.; Lista, F.; Arduini, F. Smartphone-assisted paper-based electrochemical immunosensor for SARS-CoV-2 detection in saliva. *Bioelectrochemistry* **2024**, *156*, 108619.
23. Xu, J.; Shen, J.; Zhang, B.; Zhang, Y.; Lv, X.; Zhu, G. Smartphone-based portable electrochemical-colorimetric dual-mode biosensor for glucose detection in a co-reaction system. *Electrochim. Acta* **2024**, *481*, 143952.
24. Nassar, J.M.; Mishra, K.; Lau, K.; Aguirre-Pablo, A.A.; Hussain, M.M. Recyclable nonfunctionalized paper-based ultralow-cost wearable health monitoring system. *Advanced Materials Technologies* **2017**.
